# SEMMs: Somatically Engineered Mouse Models. A New Tool for *In Vivo* Disease Modeling for Basic and Translational Research

**DOI:** 10.3389/fonc.2021.667189

**Published:** 2021-04-23

**Authors:** Anthony Lima, Danilo Maddalo

**Affiliations:** ^1^ Department of Translational Oncology, Genentech, Inc., South San Francisco, CA, United States; ^2^ Roche Pharmaceuticals, Basel, Switzerland

**Keywords:** clustered regularly interspaced short palindromic repeat/CRISPR associated protein 9-mediated genome editing, mouse models, genetically engineered mouse models, somatically engineered mouse models, translational research, animal models

## Abstract

Most experimental oncology therapies fail during clinical development despite years of preclinical testing rationalizing their use. This begs the question of whether the current preclinical models used for evaluating oncology therapies adequately capture patient heterogeneity and response to therapy. Most of the preclinical work is based on xenograft models where tumor mis-location and the lack of the immune system represent a major limitation for the translatability of many observations from preclinical models to patients. Genetically engineered mouse models (GEMMs) hold great potential to recapitulate more accurately disease models but their cost and complexity have stymied their widespread adoption in discovery, early or late drug screening programs. Recent advancements in genome editing technology made possible by the discovery and development of the CRISPR/Cas9 system has opened the opportunity of generating disease-relevant animal models by direct mutation of somatic cell genomes in an organ or tissue compartment of interest. The advent of CRISPR/Cas9 has not only aided in the production of conventional GEMMs but has also enabled the bypassing of the construction of these costly strains. In this review, we describe the Somatically Engineered Mouse Models (SEMMs) as a new category of models where a specific oncogenic signature is introduced in somatic cells of an intended organ in a post-natal animal. In addition, SEMMs represent a novel platform to perform *in vivo* functional genomics studies, here defined as DIVoS (Direct In Vivo Screening).

## Introduction

Integrative molecular analysis of human and cancer genomes has thrown a spotlight on the immense complexity of tumor biology (1). The ability to identify driver mutations and discriminate them from passenger events is key for the design of effective therapies to induce tumor regression. Moreover, being able to understand how tumors interact with the surrounding microenvironment and in particular with the immune system, is paramount for the preclinical design of therapeutic approaches combining targeted and immuno-therapy (2, 3). In this particular context, Genetically Engineered Mouse Models (GEMMs) of cancer can be invaluable in defining mechanisms driving tumor initiation, progression, and response to therapy (4). However, despite the wealth of information, they could provide a few challenges that have so far limited the use of GEMMs for preclinical drug development. First, the generation of GEMMs is time-consuming because it requires several steps such as precise gene targeting in Embryonic Stem Cells (ESCs), implantation, germline transmission, and colony expansion before achieving an experimental cohort. Timelines of this process would be further expanded when multiple allele engineering is required. Another limitation is that only a fraction of the animals employed in the generation process will eventually be used as experimental models, resulting in high husbandry costs and animal waste. Moreover, in many cases the phenotype penetrance of some oncogenes is only partial, requiring additional mice to have conclusive studies, and the tumor latency could be over a year, like in the case of animal models of squamous cell lung carcinoma (SCLC) (5). Last but not least, the ideal GEMM would develop tumors in a specific tissue as a consequence of mutations occurring in somatic cells rather than the germline. Even if this last point has been partially addressed by generating conditional models leveraging the Cre-recombinase or Tet-on inducible systems, the amount of breeding and crossing required makes the process tedious and expensive. A valid alternative to the GEMMs has been represented in the last few years by animal models where somatic cells would be directly engineered. We define as SEMMs (Somatically Engineered Mouse Models) *in vivo* models were a given genetic modification is induced in a specific somatic cell of an intended organ. Typically, somatic editing would be performed on animals with a wild type genotype using genome editing enzymes such as Cas9 or its derivatives or the Cas12a/Cpf1 delivered directly *in vivo* along with a set of specific sgRNAs. More in general, the concept of SEMM is not formally limited to rodents but can be extended to other species. The immediate advantage of SEMMs over GEMMs is the time efficiency, as almost no breeding is needed, the drastic reduction of animals required to achieve an experimental cohort as well as the versatility since multiple sgRNAs can be delivered simultaneously to mimic complex oncogenic events (for a comparison between GEMMs and SEMMs refer to **Table 1**).

**Table 1:** Comparison of GEMMs and SEMMs.

	Advantages	Disadvantages
**GEMMs**	Homogeneous genotype	ESC engineering required
	Possibility to have heterozygous or homozygous lesions	Complex breeding steps required if multiple genes are involved
	Point mutations are easily modeled	Low tumor mutation burden
**SEMMs**	Short timelines as germline engineering is skipped	Genome editing enzymes as well as delivery systems may be immunogenic
	Possibility to induce chromosomal rearrangements (inversions, translocations, deletions)	Low efficiency to introduce point mutations
	Possibility to perform directly *in vivo* screens	Low tumor mutation burden

## SEMMs: Development and Applications

Achieving precise, efficient, and consistent genome editing directly *in vivo* is feasible due to the successful combination of the CRISPR/Cas9 system with efficient delivery systems that vary according to the target organ (6). Generation of a desired oncogenic signature in a SEMM is dependent on a two-step process: first, the DNA damage induced by the endonuclease and second the consequent repair event. Introduction of a double-strand break triggers two distinct and competing repair mechanisms: the Non-Homologous-End-Joining (NHEJ) pathway results in the insertion or deletion (indels) of nucleotides at the cutting side, resulting in frameshifts (7); the Homologous Recombination (HR) pathway uses a foreign DNA template that can be incorporated at the site of damage (7). It has to be noted however, that especially *in vivo*, the NHEJ pathway is dominant over the HR mechanism and therefore strategies involving the latter should be carefully designed. Based on which mechanism is leveraged, *in vivo* delivery of the Cas9 can inactivate tumor suppressors and/or trigger mutations at hotspots on oncogenic drivers. Also, CRISPR-based models offer the unique advantage of generating large chromosomal rearrangements such as inversions, deletions, and translocations (8). It was indeed not possible to trigger such events in somatic cells *in vivo*, apart from approaches where multiple rounds of ESC engineering were required. Last but not least, SEMMs offer the unique advantage of screening directly *in vivo* for genes contributing to tumor development, and resistance in a specific organ of interest.

## SEMMs Simplify Genetic Mouse Modeling

**Figure 1:**
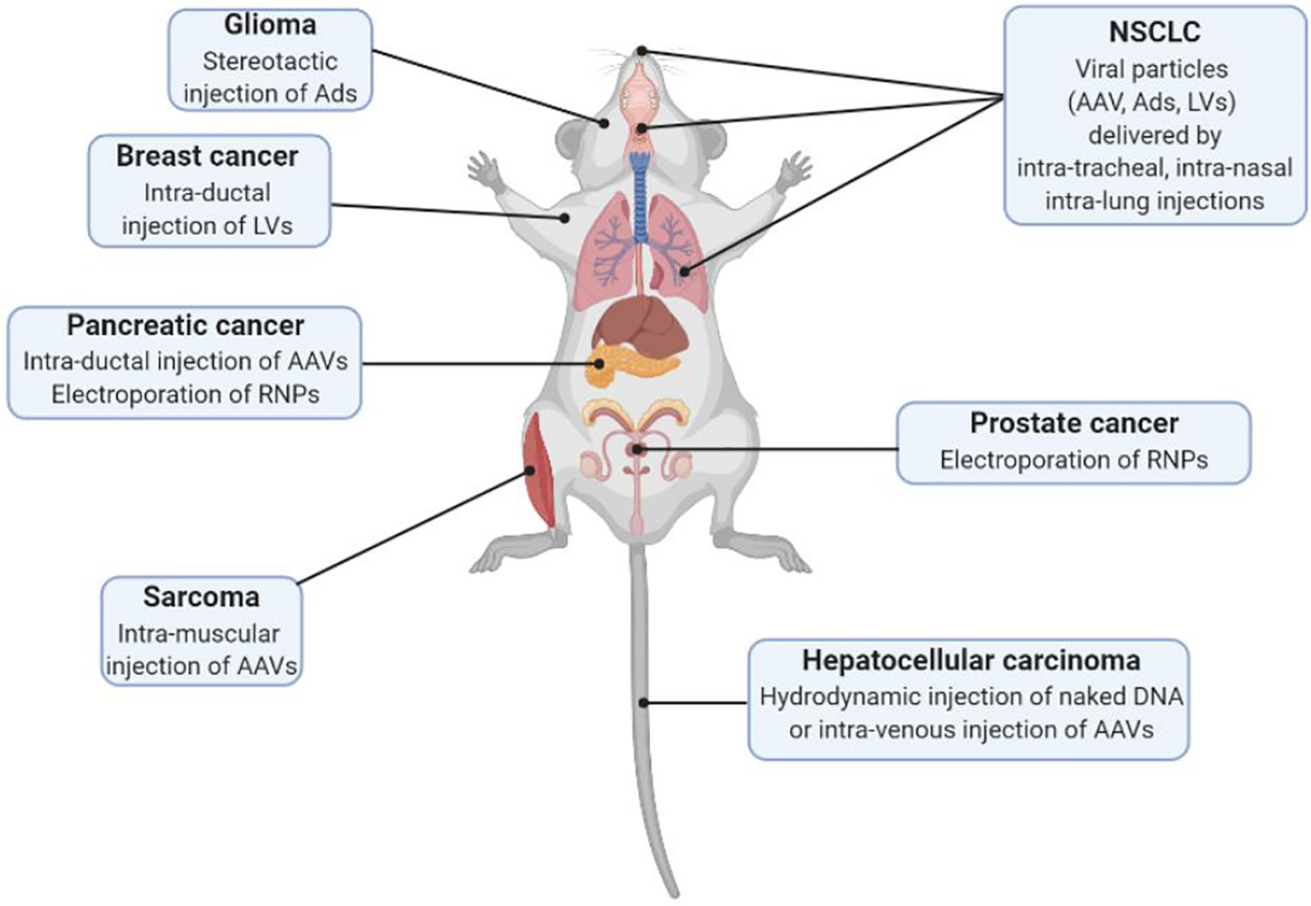
Schematic representation of different delivery modalities of the CRISPR/Cas9 system *in vivo* for SEMM generation (AAV, Adeno Associated Virus; Ads, Adenoviruses; LVs, Lentiviruses; RNPs, Ribonucleoproteins).

The possibility of delivering the CRISPR/Cas9 system *in vivo* with viral and non-viral methods (summarized in **Figure 1**) along with the generation of animal models expressing constitutively or conditionally the Cas9 endonuclease, has opened the possibility of generating various animal models in a cost- and time-efficient way. Notably, patient-derived genomic information plays a two-fold role: while on one hand it guides the generation of murine models of disease, on the other hand it is the main source of information for designing specific libraries to interrogate the genome directly *in vivo*.

At this stage the main limitation for the generation of a SEMM is the tissue tropism of a given delivery system and the possibility of editing a specific cell population. In the section below we report as proof of concept limited examples of SEMMs organized by target organ generated in the past few years (summarized in **Table 2**).

**Table 2:** Summary of SEMMs and application as DIVoS.

Organ	Tumor type	Oncogenic signature	Delivery	Route of injection	Genotype	Reference
Liver	HCC	Pten^-/-^/Trp53^-/-^/Ctnnb1 mutation	Naked DNA	Tail vein (hydrodynamic)	WT	Xue et al. (9)
	Fibrolamellar carcinoma	Dnajb1–Prkaca translocation	Naked DNA	Tail vein (hydrodynamic)	WT	Engelholm et al. (10)
	HCC	*DIVoS*	AAV	Tail vein	LSL-Cas9-eGFP	Wang et al. (11)
Lung	NSCLC	Eml4-Alk inversion	Adenovirus	Intra-tracheal	WT	Maddalo et al. (12)
		Eml4-Alk inversion	Lentivirus	Intra-pulmonary	WT	Blasco et al. (13)
		Kras^G12D^/Pten^-/-^/Trp53^-/-^	Lentivirus	Intra-tracheal	LSL-KRas^G12D^	Sanchez-Rivera et al. (14)
		KRas^G12D^/Trp53^-/-^/Lkb1^-/-^	AAVs	Intranasal	LSL-Cas9	Platt et al. (15)
		KRas^G12D^/Trp53^-/-^/Keap1^-/-^	Lentivirus	Intra-tracheal	LSL-KRas^G12D^	Romero et al. (16)
Oral cavity	HNSCC	*DIVoS*	Lentivirus	Intra-amniotic	LSL-Cas9*-*GFP	Loganathan et al. (17)
Brain	Glioma	Bkan-Ntrk1 translocation	Adenovirus	Intracranial	WT	Cook et al. (18)
	Medulloblastoma Glioblastoma	Ptch1 Trp53^-/-^/Pten^-/-^/Nf1	Naked DNA	PEI transinfection *in utero* Electroporation	WT	Zuckermann et al. (19)
	Glioblastoma	*DIVoS*	AAV	Stereotaxic	LSL-Cas9-eGFP	Chow et al. (20)
Muscle	Sarcoma	*DIVoS*	AAV9	Intramuscular	H11-LSL-Cas9	Winters et al. (21)
Pancreas	PDAC	Trp53^-/-^/Lkb1^-/-^/Arid1a^-/-^	AAVs	Intra-ductal	LSL-Cas9	Ideno et al. (22)
	PDAC	*DIVoS*	RNP	Electroporation	Ptf1a-Cre/ LSL-KRas^G12D^	Maresch et al. (23)
Breast	ILC	Pten^-/-^	Lentivirus	Intraductal	Cas9 or WT with Cdh1fl/fl/ LSL-AktE17K	Annunziato et al. (24)
Prostate	CRPC	Myc^high^/Pten^-/-^	RNP	Electroporation	WT	Leibold et al. (25)
Colon	Colorectal cancer	Kras^G12D^/Trp53^-/-^/Apc^-/-^	Lentivirus	Intra-mucosal (colonoscopy assisted)	WT or LSL-eGFP-Cas9	Roper et al. (26)

NSCLC, Non Small Cell Lung Cancer; HNSCC, Head and Neck Squamous Cell Carcinoma; HCC, Hepatocellular Carcinoma; PDAC, Pancreatic Ductal Adenocarcinoma; ILC, Intraductal Lobular Carcinoma; CRPC, Castration Resistant Prostate Cancer; RNP, Ribonucleoprotein; AAV, Adeno Associated Virus; LSL, Lox Stop Lox; WT, Wild Type; DIVoS, Directly In Vivo Screening.

### Liver

It is not surprising that the liver is among the first organs to be targeted to generate SEMMs as delivery of plasmid DNA can be easily achieved with the use of hydrodynamic injection (HDI) (27). While not a practical delivery method for clinical use, many groups found they could generate human-relevant disease mouse models using the technique (28). One of these groups combined a plasmid with an AAV integration sequence to reverse fumarylacetoacetate hydrolase (FAH) deficiency in mice and the same delivery technique was successfully used to deliver Cas9 and an sgRNA to accomplish the same treatment (a similar deficiency occurs in individuals with hereditary tyrosinemia type 1 (HT1) leading to liver failure and hepatocellular carcinoma (28). Clinical procedures for human HDI are being developed which mimic the delivery of therapeutic agents to humans with safer lower volumes of fluid and in more direct routes that target the liver or portions of the liver (29). Delivering plasmid DNA by HDI presents several advantages over a viral-based platform: first, viral packaging is not required making it safer while also making gene delivery more cost and time effective. Second, depending on the construct delivered, the CRISPR/Cas9 system is only transiently expressed, minimizing off-target effects and immunogenicity; third, it can be easily combined with other systems, such as the Sleeping Beauty or the PiggyBac transposase, for concomitant gene overexpression.

Researchers have demonstrated that CRISPR mediated knockdown of tumor suppressor genes commonly found in human cholangiocarcinoma such as Trp53, and Pten, results in tumor development within 3 months. All animals developed liver tumors with bile duct differentiation features consistent with tumors that arise from Cre-mediated double conditional knockout mice. Additionally, they demonstrated that an sgRNA targeting β-catenin (*Ctnnb1*) co-delivered with a 200-nucleotide single stranded DNA oligonucleotide could induce a gain-of-function mutation (9). Such an approach demonstrated that *in vivo* genome editing could not only induce gene knockouts but also very precise mutations by homologous recombination. However, it has to be noted that the donor integration efficiency is still very low as only around 0.4% of hepatocytes showed nuclear β-catenin 7 days after injection. Similarly, SEMMs were generated to investigate the synergistic effect of knocking out a tumor suppressor such as *Pten* and/or *Trp53* together with the expression of an oncogene such as *NRas* or *cMet* (mutated or wild type) as well as the impact on tumor latency in HBV-driven models (9).

Another approach to combine gene KO and overexpression was shown in two models of intrahepatic cholangiocarcinoma (ICC) driven by the loss of the *Trp53* gene and the expression of the oncogenes *KRas^G12D^* or *HRas^G12V^*. In this approach gene integration was achieved by a dual sgRNA approach: one sgRNA linearized the donor plasmid while the second one targeted the integration site on the genome, the 3’-UTR region of the strongly expressed β-Actin gene. Even if this method has shown some success in tumor induction, it is however limited by events such as random integration of the plasmid donor, misorientation of the construct and inconsistency in the tumor penetrance (30).

The generation of SEMMs is also an efficient method to determine the oncogenic potential of uncharacterized genetic lesions such as fusions resulting from chromosomal rearrangements. For example, a SEMM of fibrolamellar hepatocellular carcinoma (FL-HCC) was generated by triggering the intra-chromosomal deletion *Dnajb1-Prkaca*. This phenotype highlighted the oncogenic potential, *in vivo*, of the fusion DNAJB1-PRKACA and its role in the initiation of FL-HCC (10). Induction of oncogenic gene fusions *in vivo* with the CRISPR/Cas9 system has several implications. First, it simplifies remarkably disease modeling as previously resembling gene rearrangements relied upon transgene expression (31, 32) or sequential introduction of loxP sites (33, 34). Second, it represented a clear warning concerning the safety of future Cas9-based therapies where sgRNA off targets may induce chromosomal aberrations.

### Lung

SEMMs of lung cancer have been among the first models generated as the lung epithelium is easily accessible without the need for invasive surgery and highly infective by viral vectors. Lung cancer models are of particular clinical relevance since adenocarcinomas of the lung epithelium are the most frequently occurring cancers. Estimated new cases for the U.S. in 2020 alone are 228,820 or 13% of total new cases with 135,720 or 23% estimated number of U.S. deaths for 2020 (35). Sequencing data from 188 cancers has shown that the most common changes found in lung adenocarcinoma are mutations in genes such as TP53, STK11, PTEN, CDKN2A, KRAS, BRAF, EGFR, ERBB2, PIK3CA (35). The first SEMMs of NSCLC reported modeled the oncogenic mutation *KRas^G12D^* with the inactivation of tumor suppressors such as *Trp53* and *Lkb1/Stk11* (15). Platt and colleagues generated *Kras^G12D^*/*Trp53^KO^*/*Lkb1^KO^* lung tumors by delivering an AAV expressing the Cre recombinase to induce Cas9 expression, a donor template targeting exon 2 of the *Kras* gene to introduce the point mutation G12D and three sgRNA targeting *Trp53*, *Lkb1*, and *KRas* (15). When crossed with a β-actin Cre driver line, progeny mice with constitutive Cas9 expression were reported to be phenotypically normal down to a type of particularly sensitive CA1 pyramidal neuronal cells (15). This approach was the first example of a murine model expressing the Cas9 enzyme, resulting in increased versatility as viral particle capacity would otherwise be impacted by the genetic size of the endonuclease (approximately 4 kilobases). It has to be noted that such approach also highlighted the limitation of the CRISPR/Cas9 system to induce specific point mutations as the HDR pathway is significantly disadvantaged compared to the NHEJ, resulting in prolonged tumor latency or due to indels and/or incorrect inclusion of the DNA donor template. A more consistent experimental outcome was obtained when heterozygous *LSL-Kras^G12D^* mice were infected with lentivirus containing expression vectors for Cas9, Cre, and a sgRNA targeting *Trp53*. The resulting adenocarcinomas were consistent with NSCLC features demonstrated by Cre-activated lung tumors of *Kras^LSLG12D/WT^xTrp53^flox/flox^* established tumor models (15). The versatility of such a model has been shown in a parallel study investigating the role of the KEAP1 (16), PTEN, NKX1 and APC (14) in the same GEMM of KRAS-driven lung cancer. Manipulation of somatic cells by CRISPR-Cas9 mediated genetic loss can alter oncogenic pathways in an already well-established mouse genetic tumor model such as *Kras^LSLG12D/WT^xTrp53^flox/flox^*. This mouse model was used to study the effect and possible vulnerabilities of *Keap1* loss in human LUAD (16). This demonstrates the many combinations of techniques, mouse strains and targeting vectors that can be used to create useful and clinically-relevant models without the need for further genetic targeting and breeding.

Chromosomal aberrations such as deletions, inversions and translocations are frequent drivers of lung cancer and this particular type of genetic perturbation has been difficult to recapitulate in germline genetic models as it required multiple rounds of ESC engineering. For the first time genetic aberrations have been generated directly *in vivo* with the CRISPR/cas9 system. In this instance the genetic inversion EML4-ALK, found in approximately 5% of NSCLC patients, was modeled in a lung SEMM where two sgRNAs targeted the intronic region of *Eml4* and *Alk*. While *in vitro* the two double strand breaks resulted in a variety of combinations including indels, deletions and inversions, *in vivo* the fusion *Eml4-Alk* was positively selected and drove the formation of NSCL with histopathological feature overlapping with the human disease. There are two examples of generation of a SEMM of EML4-ALK inversion in the lung epithelium. In one case two sgRNAs and the Cas9 enzyme were delivered by intra-tracheal injection to the lung epithelium by an all-in-one adenoviral system, resulting in tumor formation as early as four weeks (12). In another case the same lesion was induced by two distinct lentiviral particles each expressing an sgRNA directly injected into the lung (13). Notably, in this case tumor incidence and latency were significantly less efficient, suggesting that viral tropism, vector design and relative sgRNA cutting efficiency play a crucial role in the successful design of a SEMM of chromosomal rearrangements.

### Brain

Identification of genetic lesions driving tumor formation in the brain has been elusive due to the high number and the low frequency of mutations making the use of germline models for driver and therapeutic screening time consuming and expensive. An extensive effort at characterizing 543 patients with a subset contributing to whole-exome sequencing data identifying 71 significantly mutated genes (36). Additionally, a number of fusion gene products have been identified by recently developed heuristics investigating potentially dysregulating gene altering events (37). Generation of SEMMs *via* transformed *Trp53* null adult neural stem cells implanted into immunodeficient NCr-Foxn1nu mice helped characterize four potential gene-fusion drivers: *Fgfr3-Tacc3*, *Sec61g-Egfr*, *Bcan-Ntrk1* and *Gga2-Prkcb*, but only cells harboring the fusion BCAN-NTRK1 generated high-grade gliomas (18). These tumors also responded to therapeutic inhibition with entrectinib, an inhibitor specific to the NTRK1 kinase but interestingly not through caspase mediated apoptosis.

Cook and group went on to create an autochthonous model generated by inducing an intra-chromosomal deletion *via* stereotactic intracranial injection of adenoviral particles expressing the Cas9 and two sgRNAs. Upon infection, wild type animals developed high grade glioma within 3 months with a penetrance of roughly 50%, demonstrating the ability of the fusion gene to drive tumor formation. Another approach interrogated multiple tumor suppressor genes by a low throughput loss-of-function screening to identify oncogenic events driving the initiation of medulloblastoma and glioblastoma. In this study the CRISPR/Cas9 system was delivered as expression plasmids either by in utero electroporation of (E13.5) embryos or by polyethylenimine (PEI) mediated intracranial transfection of P0 postnatal mice. These methods of transfection overcome many technical hurdles such as size limitation, immunogenicity, and costs involved in viral vector delivery (19). The blood-brain barrier (BBB) is a major obstacle to the delivery of viral and plasmid vectors that must be overcome as in previous examples by direct injection into the target area or mediated by surgery and specialized transfection techniques such as electroporation. Made up of tight-junctions in endothelial cells and other anatomical components, systemic delivery of transforming vectors to various cell types of the CNS requires overcoming this obstacle. Groups in the field of gene therapy are making progress in this area by the use of recombinant AAV of various serotypes with demonstrated capability of traversing the BBB (38). For example AAV9 has shown the ability to penetrate endothelial cell junctions in an *in vitro* model of the BBB and infect many CNS cell types, albeit at a lower transduction efficiency (39).

Investigation into the possibility of delivering a retroviral vector capable of expressing MYC to induce medulloblastoma tumors similar to MYC expressing pediatric tumors demonstrated a low penetrance but metastasizing model when coupled with either *Trp53* loss or *Bcl-2* expression (40). The group injected a cell line capable of constitutively producing virus to the brains of RCAS-TVA animals. This approach uses replication-competent avian leukosis virus splice-acceptor (RCAS) vectors to target individual cells engineered to express the cell surface receptor TVA. Cells transduced with the retrovirus will express MYC and either lose tumor suppressor expression or express the apoptosis inhibitor BCL-2 and results in a low penetrance but highly metastatic model, a marker of poor prognosis in the clinic.

### Muscle

Point mutations can be introduced into tissues using libraries of viral vectors to discover how different point mutations affect the mutant gene’s tumorigenicity in practically any tissue type (21). This platform utilized a multiplexed set of AAV targeting vectors that uniquely barcoded single-nucleotide point mutations of *Kras* exon2 and 3 to discover how different point mutations are expressed in tumors. Winters et al. used Cas9-mediated homology-directed recombination (HDR) to introduce this diverse set of uniquely barcoded *Kras* point mutations in muscle to demonstrate how different point mutants of *Kras* affect the formation of soft tissue sarcomas. This technique closely models two types of pediatric sarcoma found to harbor *Kras* mutations. Mice were injected with AAV-KRas donor/sgKras/Cre into the gastrocnemii of PT;H11^LSL-Cas9^ mice to initiate tumors. 3-7 months later 5/7 mice subjected to this procedure produced invasive sarcomas with oncogenic *Kras^G12D,^ Kras^G12A^*, or *Kras^G13R^* alleles but few tumors harbored other point mutations. Viral delivery *via* intramuscular injection is a simple procedure that allows for the relatively rapid transformation of normal muscle cells to facilitate multiplexed functional studies for the rapid identification of driver mutations *in vivo.* This group was able to couple this system with next-generation sequencing in models that produce multiple tumor foci and metastasizes to distant locations to enable the determination of lesion size, location and even the tracking of distant metastasis to the originating tumor (21).

### Pancreas

Over 48,000 new cases of pancreatic ductal adenocarcinoma (PDAC) in the United States and nearly as many deaths from this aggressive disease in 2015, PDAC is predicted to become the second leading cause of cancer mortality by 2030 (41). Models for this target organ would require survival surgery but groups with experience in the technique report high rates of success with little training of personnel (42). The production of three complex PDAC models was accomplished by AAV-mediated delivery of sgRNA into the murine pancreas of p48-Cre/LSL-Cas9 mice (22).These models recapitulate the phenotype of *Kras^G12D^* driven tumors with cooperating *Trp53*, *Lkb1* and *Arid1A* lesions in a single workhorse mouse by the injection of different AAV packaged sgRNAs that direct the pancreatic epithelium-specific p48 promoter driven Cas9. This study demonstrated how a single germline engineered mouse could produce a wide range of tumor types. Since fewer stains of mice could cover a wider panel of specific tumor types and indications, mice are more likely to be available shortening the timelines to perform *in vivo* studies. The ability to multiplex gene editing CRISPR/Cas9 sgRNAs was performed in a carefully optimized set of experiments where precisely controlled electroporation of the pancreas facilitated the uptake of injected plasmid preparations (23). The group, interestingly, discovered a negative-selection of *Brca2* inactivation in cancer cell lines derived from CRISPR/Cas9 derived PDAC tumors, demonstrating the feasibility of using this approach to explore therapeutic vulnerabilities *in vivo* (22).

### Hormone Dependent Cancers

Somatic engineering to induce malignancies driven by hormone receptors such as breast and prostate cancer has allowed deeper understanding of pathways key to tumor initiation and to the interaction with the microenvironment. Breast cancer patients are molecularly stratified based on their estrogen, progesterone and HER-2 status (43). Estrogen receptor status has been a diagnostic marker since the 1970’s and closely recorded since 1990 but is absent in mice (44). This, among other differences, between mice and human breast tissue partially explains the difficulty of engineering a genetic mouse model that recapitulates human indications even if subgroups of breast cancer make this a promising area of development for SEMMs. Breast cancers that arise in mice not only require fewer oncogenic events to form and have general chromosomal stability with rare telomere dysfunction but do contain structural rearrangement albeit fewer than human breast tumors (45). The development of new SEMMs will need to address these differences when translating results to the clinic. While a large number of effective treatments are available (46) resistance remains a major contributing factor to death from this disease. To address this problem many groups are turning to CRISPR/Cas9 systems to interrogate potential targets involved in resistance to current therapies. This approach could have benefits to patients that have developed multi-drug resistance to re-sensitize tumors. The path to this therapy requires knowledge of the precise mechanism of resistance and if that mechanism would be amenable to gene editing therapy. For example, the PI3K pathway has been shown to confer partial resistance to anti-HER2 therapy in a HER2 positive and PIK3CA^H1047R^ mouse model and organoid (mammosphere) system derived from the strain (47). This system lends itself to discovery of the resistance mechanism in organoids using a CRISPR/Cas9 discovery platform to find novel resistance marker first in the organoids then verify hits *in vivo* in the mouse strain using a targeted sgRNA (47). In another report it was also shown how intra-ductal lobular breast carcinoma (ILC) formation was impacted by Cas9-driven immunogenicity, in a mouse model where Cre mediates inactivation of *E-cadherin1* (*Cdh1*) and activation of *Akt^E17K^*. While intraductal injection of lentiviruses encoding Cre, Cas9 and an sgRNA targeting Pten resulted in highly infiltrated tumors not resembling the human disease, delivery of a lentiviral vector encoding the Cre and the same sgPten in a model expressing constitutively the Cas9 efficiently induced ILC (24). In another work it has been shown that electroporation of the prostate gland with the complex Cas9/sgRNAs is able to induce prostate cancer *in vivo* with tumor latency and penetrance comparable to the traditional GEMMs (25). Once again, this SEM model, defined by the authors as EPO-GEMM, provided a time- and cost- efficient approach to generate complex genotypes and identify the Wnt pathway as a key regulator of metastases.

### Colon

Many GEMM models of colorectal cancer (CRC) suffer from lack of tumor foci location and frequency control. APC GEMM models only develop tumors in the small intestine quickly occluding the intestinal tract before any develop to carcinomas and metastasize. Colorectal cancers are currently best recapitulated with their low number of foci of primary tumors, location in the colonic submucosa, and metastatic behavior to distant organs, most commonly, liver and the lung with or without the use of GEMM model mouse strains by utilizing colonoscopy (26). In a detailed paper Jatin et al. (2018) lay out procedures to study colon cancer in mouse model systems. CRISPR/Cas9 is used to edit organoids grown and edited *in vitro* for colonoscopy guided implantation into the colon submucosa. This technique could also be used to directly insert CRISPR/Cas9 viral or DNA vectors capable of editing the genome of target cells. Colonoscopy allows researchers to reach the distal colon and avoid transplantation or induction of the rectum, which is more difficult and traumatic to the mouse if attempted with rectal prolapse. Colonoscopy while costly in equipment investment is indispensable for CRC research as it allows for the targeted induction of models, minimally invasive surveillance of tumor development and tumor sampling and avoids mortality of subject mice experienced from survival surgery techniques. Coupling colonoscopy and somatic gene editing can greatly reduce the number of animals used for CRC research. Research looking at the most common mutated genes in CRC *APC, TP53, SMAD4, PIK3CA* and *KRAS* can be easily augmented with more recently discovered mutated genes including *ARID1A*, *SOX9* and *PAM123B* (48) using the rapid generation of relevant models.

## SEMMs as a Platform for DIVoS (Direct *In Vivo* Screening)

**Figure 2:**
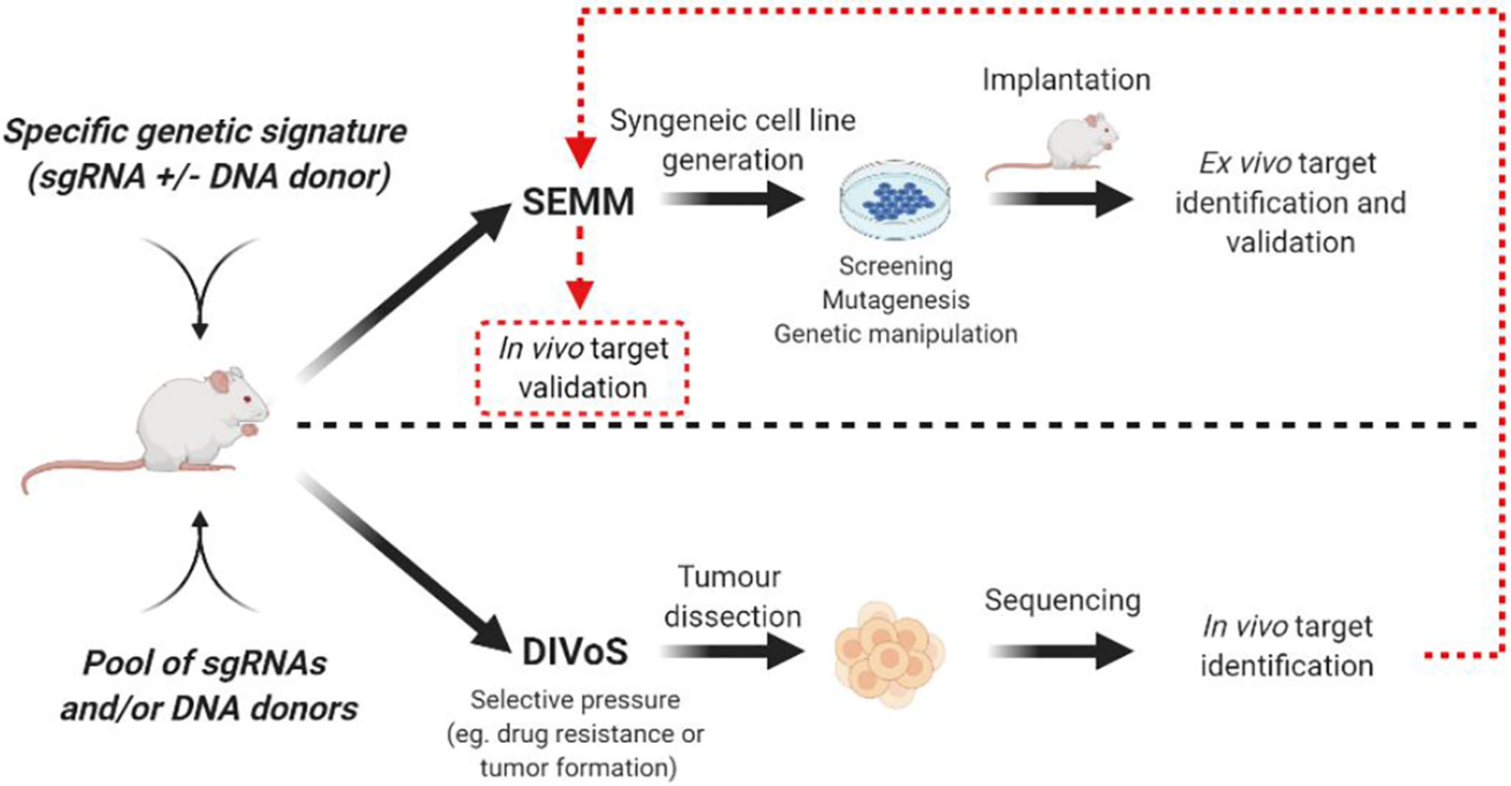
Representative workflow for SEMM generation and DIVoS design starting from CRISPR/Cas9 *in vivo* delivery. Targets identified with DIVoS are eventually validated with the generation of a SEMM (red dotted line).

In addition to the immediate advantage of time and cost savings over the GEMMs, SEMMs hold the promise of playing an important role to perform Direct *In Vivo* Screening (DIVoS) enabling the discovery of genes and/or gene networks relevant only in a specific context and environment. Ideally, genes identified with DIVoS could be ultimately validated *in vivo* with a dedicated SEMM (**Figure 2**). Compared to classic ex-vivo screening, an approach of screening directly *in vivo* has several advantages. First, it allows the interrogation of genes (especially tumor suppressors) key to tumor initiation and influencing sensitivity to therapy. In addition, the *in vivo* target cell population is surrounded by its original microenvironment compared to cell cultures, making data interpretation more straight forward as it is not influenced by culture conditions such as media and 2D vs 3D cell seeding. Moreover, since the screening takes place concomitantly to tumor formation, specific oncogenic signatures can be chosen upfront. In addition, DIVoS performed using SEMMs as a platform are clearly distinguished from previous efforts to perform screening *in vivo* using transposon systems such as Sleeping Beauty or PiggyBac, as in this case genome interrogation is random and limited to specific sequences (49, 50). As one of the major hurdles for this approach is the limited take rate due to immune-rejection of the viral vectors utilized to deliver the libraries, it is not surprising that only a specific pool of genes can be investigated for any given screening campaign and that gain-of-function approaches are highly favored compared to drop out screens. Also, organ accessibility and viral tropism play a key role in determining which tumor types can be easily screened. The generation of animal models expressing the Cas9 endonuclease and the refinement of viral vectors has built the core of a platform to screen, *in vivo*, hundreds of genes by sgRNA infection. The typical workflow for a DiVoS contemplates the identification of specific genetic modifications from a given patient population and the subsequent interrogation of such a gene pool in an *in vivo* model. One of the first example of DiVoS was the screening of 13 tumor suppressor genes electroporated into the murine pancreas where a negative selection was shown when Brca2 gene was lost. Other experiments were performed in autochthonous models of hepatocellular carcinoma (11) and glioblastoma (20) where AAV-CRISPR vectors were used to map tumor suppressor genes contributing to cancer development. In both instances it is also clear that a few technical hurdles are still limiting the screening efficiency, such as partial integration of viral vector sequences, rejection by the immune-system and generation of a multifocal lesions sometimes difficult to dissect. An elegant strategy to circumvent immune rejection used ultra-sound guided intra-amniotic injection into the surface epithelium of mice to identify long tail genes having a tumor suppressor function in head and neck squamous cell carcinoma (HNSCC) (17). Another interesting approach is also represented by a hybrid system where sgRNA are delivered to the liver by using the PiggyBac transposon system (51). This example of hybrid approach Cas9/Transposon allows *in vivo* screening campaigns requiring a significant lower amount of resources compared to viral-based technology, even if the number of genes that can be screened remains limited. It has to be noted that even if DIVoS provide an invaluable source for novel target identification, partial vector integration as well as limited take rate due to immune rejection still represent a hurdle for the development of the field. In addition, with DIVoS it is not possible to control the multiplicity of infection (MOI) as tightly as *in vitro* and a translation from mouse to human of each finding is always required (but not always possible). A summary of advantages and disadvantages of DIVoS is shown in **Table 3**. Next generation viral vectors unable to trigger an immune response will probably be a valuable tool to refine quality and resolution of these experiments.

**Table 3:** DIVoS: advantages and disadvantages.

Advantages	Disadvantages
Possibility to identify genes at the interface tumor-microenvironment	Immunogenicity limits take rate and library size
Screen is not dependent on culture conditions	Mouse to human translation is required
Cells are in 3D and surrounded by the proper environment	MOI is difficult to control

## Future Perspective and Limitations

The possibility of efficiently delivering the CRISPR/Cas9 tool *in vivo* together with the development of additional Cas9 variants to modulate gene expression and/or epigenetic status, is already offering the unprecedented opportunity to quickly test and validate biological questions and hypotheses. Such democratization will have an immediate impact on three main aspects: a) reduction of number of animals needed to generate an experimental cohort; b) reduction of the turnaround time to validate gene function in complex disease models; c) possibility to have a multiplexed approach for the interrogation of genes directly *in vivo*. We foresee that especially the last aspect will enable the discovery of novel targets, highly dependent on the context where tumors are generated and not always and necessarily supported by simple genetic analysis.

In spite of many clear advantages, it has however to be noted that SEMMs show also some limitations. First, the expression of the Cas9 protein or any endonuclease could be a source of immunogenicity, making challenging the interpretation of studies investigating the interaction between the tumor and the immune system. In addition, even when animals expressing the endonuclease are used, delivery of sgRNAs by viral vectors would again induce an immune response. Such limitation, common also in gene therapy experiments, can be overcome by alternative delivery systems using hypotonic solutions or electroporation to deliver the ribonucleoprotein complex (52). Moreover, while the CRISPR/Cas9 allows control over the induction of double strand breaks in precise areas of the genome, not much leverage is however given in terms of controlling the pathways involved in the subsequent DNA repair. Since non-homologous end joining is significantly dominant over homologous recombination, attempts to introduce specific point mutations by providing an oligonucleotide donor have proven challenging. While such option is formally viable, the low frequency and the mixed outcome in terms of editing are still a clear hurdle making germline engineering still a preferred strategy over somatic editing. In addition, with the SEMM approach is not possible to control how many alleles to edit, a crucial aspect in some studies investigating heterozygous versus homozygous gene loss. It has to be considered that, just like the GEMMs, SEMMs do not develop tumors harboring a high number of mutations and are therefore not fully representative of cancer complexity and heterogeneity. Moreover, it is sometimes difficult to engineer tissues because of the limited tropism of some viral vectors, even if evolution of AAV serotypes along with the use of tissue specific promoters can help circumventing this problem. In addition it has to be considered that each organ generally requires a specific delivery method/procedure, each of them having advantages and limitations. Last but not least, due to tumor accessibility and complexity, SEMMs will not replace (at least fully) classic xenograft models to study pharmacokinetic, pharmacodynamics and efficacy correlation of candidate molecules but could however represent a valid source of novel, more representative syngeneic murine cancer lines.

In conclusion, with increasing delivering technologies as well as the discovery of alternative endonucleases, the field of the SEMMs has the potential to expand beyond disease modeling and support/address key scientific questions in the field of functional genomics and target identification.

## Author Contributions

DM designed and conceived the original idea of the article. DM and AL wrote the article and reviewed the relevant literature. All authors contributed to the article and approved the submitted version.

## Conflict of Interest

AL was employed by Genentech, Inc. and DM was employed by Genentech, Inc. and Roche Pharmaceuticals.
